# Rapid identification of candidate genes for resistance to tomato late blight disease using next-generation sequencing technologies

**DOI:** 10.1371/journal.pone.0189951

**Published:** 2017-12-18

**Authors:** Ramadan A. Arafa, Mohamed T. Rakha, Nour Elden K. Soliman, Olfat M. Moussa, Said M. Kamel, Kenta Shirasawa

**Affiliations:** 1 Plant Pathology Research Institute, Agricultural Research Center, Giza, Egypt; 2 Department of Horticulture, Faculty of Agriculture, University of Kafrelsheikh, Kafr El-Sheikh, Egypt; 3 Department of Plant Pathology, Faculty of Agriculture, Cairo University, Giza, Egypt; 4 Department of Frontier Science, Kazusa DNA Research Institute, Chiba, Japan; Agriculture and Agri-Food Canada, CANADA

## Abstract

Tomato late blight caused by *Phytophthora infestans* (Mont.) de Bary, also known as the Irish famine pathogen, is one of the most destructive plant diseases. Wild relatives of tomato possess useful resistance genes against this disease, and could therefore be used in breeding to improve cultivated varieties. In the genome of a wild relative of tomato, *Solanum habrochaites* accession LA1777, we identified a new quantitative trait locus for resistance against blight caused by an aggressive Egyptian isolate of *P*. *infestans*. Using double-digest restriction site–associated DNA sequencing (ddRAD-Seq) technology, we determined 6,514 genome-wide SNP genotypes of an F_2_ population derived from an interspecific cross. Subsequent association analysis of genotypes and phenotypes of the mapping population revealed that a 6.8 Mb genome region on chromosome 6 was a candidate locus for disease resistance. Whole-genome resequencing analysis revealed that 298 genes in this region potentially had functional differences between the parental lines. Among of them, two genes with missense mutations, Solyc06g071810.1 and Solyc06g083640.3, were considered to be potential candidates for disease resistance. SNP and SSR markers linking to this region can be used in marker-assisted selection in future breeding programs for late blight disease, including introgression of new genetic loci from wild species. In addition, the approach developed in this study provides a model for identification of other genes for attractive agronomical traits.

## Introduction

Plants suffer from many biotic and abiotic stresses [1], which reduce quantity and quality of crop production worldwide. Late blight disease is caused by the hemibiotrophic oomycete *Phytophthora infestans* (Mont.) de Bary, one of the most destructive plant pathogens. *Phytophthora infestans* is well known as the causative agent of the Great Famine in Ireland between 1845 and 1852, which devastated potato production (*Solanum tuberosum*) [2]. After potato, tomato (*S*. *lycopersicum* L.) is the second most agriculturally important crop in the Solanaceae family. The annual global productivity of tomato has increased dramatically, to 170 million tons in 2014 [3]. However, tomato can also be damaged by the late blight disease, particularly in cool temperatures, high relative humidity (RH), and rainy or foggy conditions [4], resulting in 100% economic losses in open fields and greenhouses.

Tomato has been used in molecular genetic and genomic studies as a model for fruiting plants [5] because of its compact genome (~950 Mb) and the simple diploid genome composition of family Solanaceae. The genome sequence of tomato [6] has enabled discovery of genome-wide single-nucleotide polymorphisms (SNPs) and development of advanced molecular markers [7–10]. Although the genetic diversity of the cultivated tomato is limited [11], its wild relatives *S*. *pennellii*, *S*. *habrochaites*, *S*. *peruvianum*, and *S*. *pimpinellifolium* have many useful traits potentially applicable to improvement of the agricultural varieties. Therefore, introduction of wild tomato species into tomato breeding programs could facilitate development of new tomato lines [12–15]. Indeed, five race-specific resistance (R) genes that confer various levels of resistances against *P*. *infestans* isolates *Ph-1*, *Ph-2*, *Ph-3*, *Ph-4*, and *Ph-5* have been identified [16–22] and applied to molecular breeding by marker-assisted selection (MAS) [20]. However, a serious problem in breeding by interspecific crossing is linkage drag, in which undesirable traits linked to target traits in the wild relatives are introgressed in elite cultivars [23, 24].

In the genomics era, advanced molecular markers and genotyping technologies have helped to solve this problem [25, 26]. Simple sequence repeat (SSR) markers are useful for genomics and breeding in tomato [27–29]; however, analysis of large numbers of genome-wide SSR markers across multiple samples, such as breeding materials, is time-consuming and laborious. However, next-generation sequencing (NGS) technologies, including high-throughput sequencing and sophisticated bioinformatics techniques, can overcome these limitations. Restriction site–associated DNA sequencing (RAD-Seq) [30–32] and an alternative technique, double-digest RAD-Seq (ddRAD-Seq) [33], can skim through the genome with low cost and high throughput. These methods can be successfully implemented in gene mapping, including quantitative trait locus (QTL) analysis and genome-wide association studies (GWAS), of a vast array of crops [32, 34–38]. On the other hand, whole-genome resequencing (WGRS) enables prediction of the effects of sequence variants on gene function throughout the genome [39–43]. Therefore, a combination of RAD-Seq and WGRS analysis represents a powerful strategy for rapidly identifying candidate genes responsible for traits of interests.

Development of new tomato lines with resistance to late blight disease would be a straightforward, effective, and environmentally safe approach to managing late blight disease. Therefore, in this study, we aimed to identify map positions of genetic loci derived from a wild tomato relative, *S*. *habrochaites* that control resistance to late blight disease caused by *P*. *infestans*. We applied a ddRAD-Seq pipeline that we developed in a previous study [33] to genetic mapping of the resistance loci, and then we used a WGRS strategy to predict candidate genes for late blight disease resistance.

## Materials and methods

### Plant materials

A cultivated tomato (*S*. *lycopersicum*), Castlerock, and its wild relative, *S*. *habrochaites* (LA1777), were used in this study. Castlerock was chosen because it is susceptible to late blight disease, and LA1777 was selected because it is resistant to the Egyptian *P*. *infestans* population, as shown in a previous study by our group [15]. Seeds of Castlerock and LA1777 were provided by the Horticulture Research Institute, Agricultural Research Center (ARC), Egypt, and the Tomato Genetic Research Center (TGRC), Davis, CA, USA, respectively. An F_2_ population (n = 344) was generated from an interspecific cross between Castlerock and LA1777.

### Isolation and purification of *P*. *infestans* isolate

Isolation of the *P*. *infestans* population was conducted by placing host infected tissues under organic potato slices in converted Petri dishes containing water agar and incubating at 18°C for 7–10 days. Sporangia were picked from the abundant sporulation on the top of the slices and transferred directly onto the recommended media. Rye sucrose agar (RSA) medium [44] (60 g of rye grains, 20 g of sucrose, and 20 g of agar per liter) was used for isolation, growth, and maintenance of *P*. *infestans* isolates. Pure culture of *P*. *infestans* was conducted on rye slants at 18°C, and the cultures were preserved as a stock for further studies. *P*. *infestans* isolate EG_12 was selected from the stock of the Plant Pathology Research Institute, ARC, which was overcome five tomato genotypes containing R genes (*Ph-1*, *Ph-2*, and *Ph-3*) as well as Super Strain B, a susceptible tomato cultivar control based on virulence test [15].

### Inoculum preparation and late blight assessment

Seeds of F_2_ progeny and the parental lines Castlerock and LA1777, as well as the susceptible control (cv. Castlerock), were sown in 209 cell seedling trays with peat moss–vermiculite mixture (1:1 volume) in a greenhouse (25 ± 2°C, 16/8 h day/night). Plants were watered and fertilized regularly with N:P:K 19:19:19, and all traditional agricultural transactions were applied to maintain the plants under appropriate and healthy conditions. Eight weeks after sowing, all trays were moved from the greenhouse to growth room at the Plant Pathology Research Institute, ARC, for artificial inoculation with *P*. *infestans* EG_12 and late blight assessment.

Inoculum preparation of isolate EG_12 was performed as described [15]. Prior to artificial inoculation, the suspension was chilled at 4°C for 2–4 h [45] to allow cleavage of sporangia and release of zoospores.

After inoculum preparation, the conditions in the growth room were adjusted to 20±2°C and 100% RH for 48 h in darkness, followed by 20°C, up to 90% RH [46], and 10/14 h day/night for 10 days. All tested plants were hand-sprayed with an atomizer to cover all parts of the foliage and kept in a growth room under the conditions described above. The plants were wrapped with a plastic sheet to keep RH above 90%. F_2_ plants were evaluated individually for late blight disease at 10 days post inoculation (dpi) by visually scoring disease severity according to a numerical rating (0–6) as described [47] with some modifications: 0, immune; 1, highly resistant; 2, resistant; 3, moderately resistant; 4, moderately susceptible; 5, susceptible; 6, highly (91–100%) susceptible. All inoculated plants were scored when the susceptible control exhibited 100% disease severity (complete death).

### DNA extraction and sequencing analysis

Total genomic DNA was extracted from young leaves of the two parents and the F_2_ progeny using the DNeasy Plant Mini Kit (Qiagen Inc., Hilden, Germany). Genotypes were analyzed using ddRAD-Seq technology with the restriction enzymes *Pst*I and *Msp*I (S1 Table). The ddRAD-Seq libraries were constructed and sequenced on a HiSeq 2000 platform (Illumina, San Diego, CA, USA) in paired-end 93 bp mode as described [33].

The two parents were further subjected to WGRS. Paired-end sequencing libraries with an insert size of 600 bp were prepared as described [48]. The nucleotide sequences were determined using massively parallel sequencing by synthesis on an Illumina HiSeq2000 (Illumina) in paired-end 93 bp mode.

### Computational data processing and association analysis

Primary data processing of ddRAD-Seq and WGS sequence reads was performed as described in our previous studies [33, 49] with some modifications. Low-quality sequences were removed and adapters were trimmed using PRINSEQ (version 0.20.4) [50] and fastx_clipper in the FASTX-Toolkit (version 0.0.13) (http://hannonlab.cshl.edu/fastx_toolkit). The filtered reads were mapped onto tomato genome SL3.0 [6], used as a reference sequence, with Bowtie 2 (version 2.1.0; parameters:—minins 100—no-mixed) [51]. The resultant sequence alignment/map format (SAM) files were converted to binary sequence alignment/map format (BAM) files and subjected to SNP calling using the mpileup option of SAMtools (version 0.1.19; parameters: default) [52] to yield a variant call format (VCF) file including SNP information. Moreover, to obtain high-confidence SNP markers, VCF files were filtered with VCFtools (version 0.1.14) [53]. The parameters for VCFtools were as follows:—maf 0.05—max-alleles 2—min-alleles 2—minDP 10—minQ 10—non-ref-ac 2—max-non-ref-ac 2—max-missing 0.75 for WGRS data; and—remove-indels—minDP 5—minQ 20—max-missing 1—min-alleles 2—max-alleles 2 for ddRAD-Seq data. Annotations of SNP effects on gene functions were predicted using SnpEff (version 4.2) [54]. The association analysis between phenotype and genotype data was performed using the generalized linear model (GLM) of trait analysis by association, evolution, and linkage (TASSEL) version 5.2.33 [55].

### SSR marker analysis

A total of 13 expressed sequence tag (EST)-derived SSR markers (TES markers) and ten genome-derived SSR markers (TGS markers) (S2 Table) were selected from the candidate genome regions on chromosome 6 for late blight resistance, as described in the Kazusa Marker Database (http://marker.kazusa.or.jp) [29]. These markers were used for polymorphic analysis.

### Data availability

Nucleotide sequence data for the ddRAD-Seq and WGRS analyses are available in the DDBJ Sequence Read Archive under accession numbers DRA005972 and DRA005973.

## Results

### Phenotypic assessment of disease response for the F_2_ population

**Fig 1:**
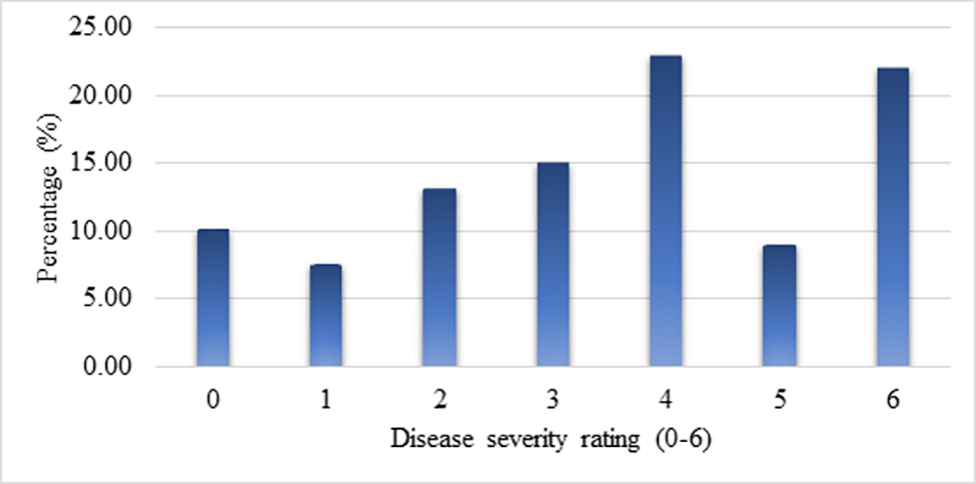
Disease severity rating 0–6 of F_2_ mapping population (n = 344) of the cross cv. Castlerock (*S*. *lycopersicum*) x *S*. *habrochaites* accession LA1777 to aggressive Egyptian isolate of *P*. *infestans*.

**Fig 2:**
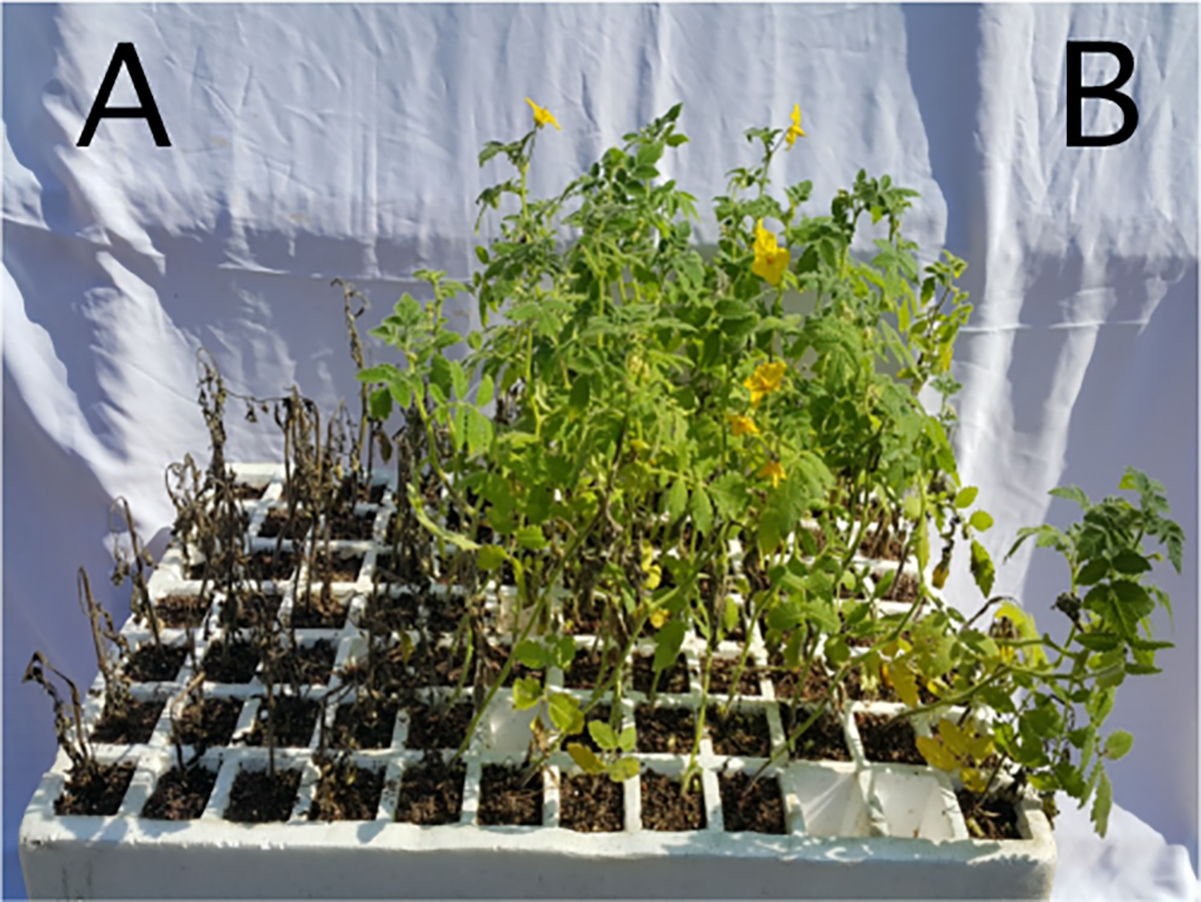
Screening the parental lines for resistance to *P*. *infestans* isolate EG_12 using whole-plant assay under controlled conditions. (A) Highly susceptible parent cv. Castlerock, (B) highly resistant parent *S*. *habrochaites* accession LA1777.

To identify QTLs associated with late blight resistance, an F_2_ mapping population of 383 plants, as well as the susceptible and resistant parents, were infected with an Egyptian isolate of *P*. *infestans* EG_12, and disease severity was evaluated on a numerical scale (0–6). All tested materials were individually scored 10 days after artificial inoculation, when the susceptible control plants reached the highest score of disease severity. The evaluated population was divided into seven categories based on the scale. The F_2_ population exhibited broad variations in reaction to the pathogen, ranging from complete resistant (0) to highly susceptible (6). In addition, varying degrees of disease severity were detected in all tested plants. Among the F_2_ population, a disease severity score of 4 was most prevalent (79 plants, 22.97%), followed by score of 6 (76 plants, 22.09%). On the other hand, a score of 1 (highly resistant) was least prevalent (26 plants, 7.56%) (Fig 1). Also, the whole-plant assay under environmentally controlled conditions confirmed that the parent *S*. *habrochaites* accession LA1777 was resistant, whereas the cultivated tomato cv. Castlerock was highly susceptible, with severe late blight symptoms (completely blighted, 100%) (Fig 2). Therefore, the tomato wild accession LA1777 should be considered a genetic resource for identification of QTLs associated with late blight resistance.

### Association analysis with SNPs based on ddRAD-Seq

**Fig 3:**
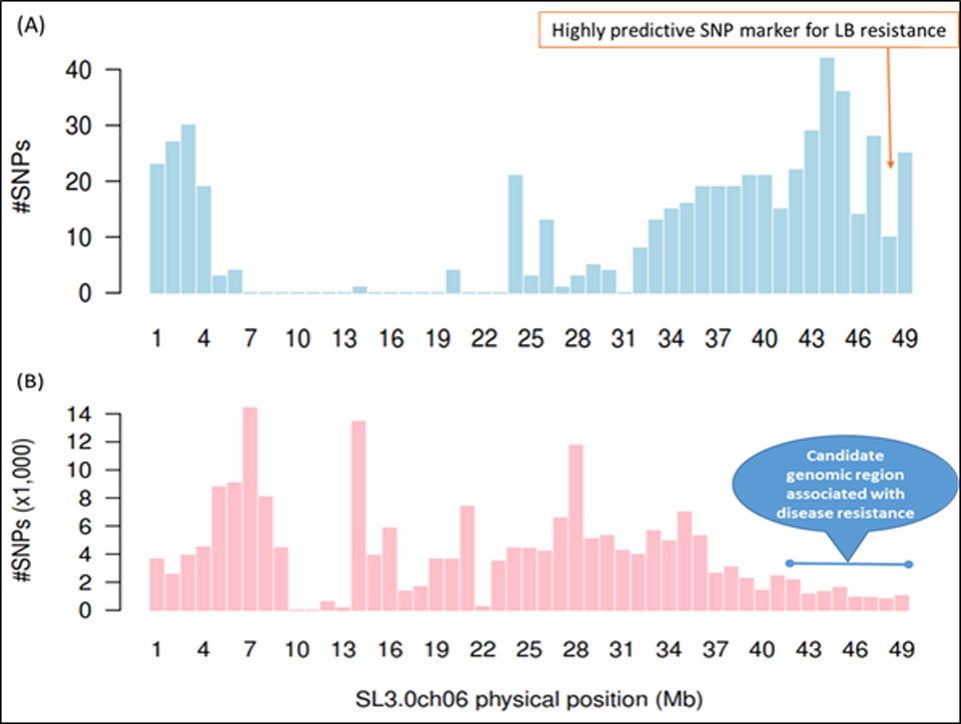
Representation of high-confidence single nucleotide polymorphism (SNP) markers along chromosome 6 of tomato mapped on SL3.0 version of the tomato reference genome. Candidate genomic region tightly related to plant disease resistance was predicted on ch06 based on SnpEff annotation, (A) the double-digest restriction site–associated DNA sequencing (ddRAD-Seq), and (B) the whole-genome shotgun resequencing (WGRS) technologies. The remaining chromosomes ch00 –ch12 are shown in S1 Fig.

In the ddRAD-Seq analysis of the parental lines and a subset of the F_2_ population (n = 150), a mean of 616,763 reads was obtained for each sample. The total numbers of high-quality paired reads of the parental lines, cv. Castlerock and *S*. *habrochaites* accession LA1777, were 1,010,157 and 367,193, respectively (S3 Table). The read numbers obtained in this study is enough for the following linkage analysis [33]. The alignment rate to the reference tomato genome build SL3.0 was approximately 90.0% in the F_2_ population, whereas those of the two parents were 93.4% (Castlerock) and 88.99% (LA1777). From the alignment data, 11,348 SNP candidates were obtained, of which 6,514 were selected as a high-quality data set (S4 Table) based on criteria described in Materials and Methods. The mean number of SNPs per chromosome (excluding 17 SNPs on sequences unassigned to the tomato chromosomes) was 543, with a variant rate of one SNP every 123,921 bases, ranging from 354 SNPs on chromosome 9 (1 SNP/205,950 bp) to 780 on chromosome 2 (1 SNP/71,766 bp). The SNPs comprised 3,406 downstream gene variants following 3,374 intron variants and 2,371 upstream gene variants. The physical positions of the 6,514 SNPs were distributed over all 12 chromosomes (Fig 3 and S1 Fig), but the distribution patterns were highly biased: most of the SNPs were located at both ends of each chromosome, which are gene-rich euchromatic regions; an exception to this pattern is chromosome 2, which has repetitive rDNA sequences at the top of the chromosome.

**Fig 4:**
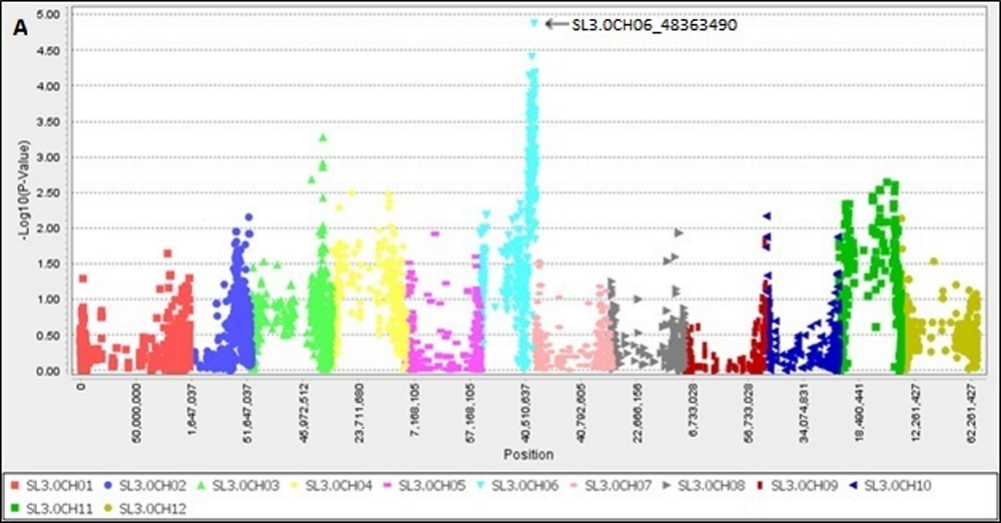
Manhattan plots for genome-wide association studies of generalized linear model (GLM) analysis of late blight disease resistance using TASSEL software. The SNP markers were generated using NGS technology, double-digest restriction site–associated DNA sequencing (ddRAD-Seq).

To detect genetic loci for resistance to *P*. *infestans* isolate EG_12, GWAS were performed with 6,514 high-confidence SNPs from the ddRAD-Seq and phenotypic data. Based on GLM with false discovery rate (FDR) of 0.1 [56], 124 SNPs on a 6.8 Mb region of chromosome 6 (42,859,404 bp to 49,665,578 bp), including 665 predicted genes, were significantly associated with phenotypic variation. Among those, the SNP at 48,363,490 bp on chromosome 6 exhibited the highest association with late blight disease resistance (Fig 4).

### Validation of the associated loci by SSR marker analysis

To validate the results of the association studies, we subjected the remaining F_2_ lines (n = 194) not analyzed with the ddRAD-Seq to genotyping analysis with 23 SSR markers that were physically and genetically close to the candidate region (S2 Table). Out of the 23 SSRs, 5 markers (TES0422, TES0014, TES1344, TES0945, and TES0213) exhibited polymorphism between the parental lines, Castlerock and LA1777 (Table 1). Therefore, we analyzed the genotypes of the additional 194 lines, as well as the 150 lines used for ddRAD-Seq, using the five selected SSR markers. As expected, the phenotypes of F2 lines with homozygous alleles from LA1777 or Castlerock differed significantly (resistant in the case of LA1777 alleles, and susceptible in the case of Castlerock alleles), even though severe segregation distortion that is often reported in intercrossing populations [29] and references therein was observed in this locus. This additional SSR analysis confirmed the results of the GWAS using ddRAD-Seq technology.

**Table 1:** Genotyping of F_2_ mapping population with five EST-SSR markers.

SSR marker	Chromosome	Position (bp)^1^	Scale^2^	Allele			Amplified samples	Total tested samples
				LA1777	Castlerock	Hete.		
TES0422	SL3.0ch06	44975890	0	17	0	13	317	344
			1	12	0	11		
			2	20	0	18		
			3	14	0	33		
			4	26	2	47		
			5	7	1	22		
			6	20	8	46		
Mean^3^				3.0431^b^	5.5455^a^	3.7895^c^		
TES0014	SL3.0ch06	45297826	0	23	0	12	343	344
			1	15	0	11		
			2	27	1	17		
			3	19	0	33		
			4	31	0	48		
			5	7	1	23		
			6	22	10	43		
Mean^3^				2.8958^b^	5.5833^a^	3.7914^c^		
TES1344	SL3.0ch06	45438555	0	23	0	12	340	344
			1	14	1	11		
			2	27	1	17		
			3	18	1	32		
			4	30	0	46		
			5	7	1	23		
			6	24	9	43		
Mean^3^				2.9441^b^	5.0000^a^	3.7935^c^		
TES0945	SL3.0ch06	47342901	0	25	1	7	328	344
			1	15	0	11		
			2	26	1	16		
			3	17	2	32		
			4	34	0	41		
			5	7	0	22		
			6	23	8	40		
Mean^3^				2.9048^b^	4.6667^a^	3.8639^a^		
TES0213	SL3.0ch06	49713763	0	24	2	9	343	344
			1	17	0	9		
			2	23	1	21		
			3	20	1	31		
			4	35	0	44		
			5	10	2	19		
			6	23	8	44		
Mean^3^				2.9671^b^	4.5000^a^	3.8362^a^		

^1^ The position based on the tomato reference genome SL3.0 version

^2^ The disease severity rating (DSR) to assessment the phenotype of late blight disease on tomato plants

^3^ Means followed by the same letter are not significantly different at *P* < 0.05 (LSD test).

The superscripts of "a", "b", and "c" are alphabetical codes indicating significant differences when the letters are different.

### Whole-genome shotgun resequencing

To identify sequence variations in the candidate genetic locus, we performed WGRS analysis on the parents. Totals of 174.9 and 189.9 million high-quality reads (17-18x genome coverage) for Castlerock and LA1777, respectively, were obtained and mapped onto the reference genome sequence, with alignment rates of 96.9% for Castlerock and 70.7% for LA1777 (S5 Table).

Across the genome including “chromosome 0”, genome sequences not assigned to any chromosomes, we identified a total of 4,180,666 high-quality sequence variations (one sequence variation every 198 bp), including 4,022,951 SNPs and 157,715 indels. The ratio of transitions/transversions (Ts/Tv) was calculated to be 1.08. The SNPs were positioned on all tomato chromosomes without large gaps (Fig 3 and S1 Fig), as observed for the genome positions of SNPs detected by ddRAD-Seq. Among the 4,180,666 sites, 14,755 (0.27%) sequence variations in 2,557 genes were predicted by the SnpEff software to possess high-impact (e.g., nonsense or frame-shift mutations) on gene functions, whereas 57,390 (1.038%) polymorphisms in 15,934 genes were predicted to have moderate impacts (e.g., missense mutations) (S6 Table).

On the other hand, in the 6.8 Mb candidate locus on chromosome 6, we identified 8,367 polymorphic sites (7,684 SNPs and 683 indels) at 1 variation/814 bases with a Ts/Tv ratio of 1.36. Among the 8,367 sites, 168 (0.87%) sequence variations in 24 genes were predicted to have high impacts, and 516 (2.67%) polymorphisms in 274 genes were predicted to have moderate impacts. In the candidate regions, the ratio of high-impact variations versus moderate-impact variations was 3-fold higher than in the genome overall, whereas variation density was lower. Among them, two genes located in the interval between the significant SNPs were considered as potential candidates for blight disease resistance genes. One was Solyc06g071810.1 encoding the leucine-rich repeat (LRR) receptor–like serine/threonine-protein kinase FEI 1 having a missense mutation at the 39^th^ position (Asp in Castlerock, Glu in LA1777), while the other was Solyc06g083640.3 for a LRR family protein with a missense mutation at the 111^th^ position (Gln in Castlerock and Lys in LA1777).

## Discussion

In this study, we identified a resistance locus for late blight disease on chromosome 6 of tomato. This locus is at a different genome position than previously reported resistance loci [22, 57, 58], and should therefore be considered novel. The result of GWAS was validated by the SSR analysis of the additional F2 lines (Table 1). In general, to confirm the accuracy of the genetic analysis of GWAS and QTL analysis, the results are validated by genotyping with DNA markers in the candidate regions. Three types of plant materials are potentially used for the validation: 1) an additional biparental population derived from the same crossing in the genetic analysis (as in this study); 2) near-isogenic lines (NILs) having target loci of the donor (e.g., a wild relative) with genetic background of the recurrent line (e.g., a cultivated line); and 3) a group of genetically divergent lines like natural populations or core collections maintaining genetic diversity of genetic pools. Among them, NILs would be the most useful materials to investigate the effects of the candidate locus on the phenotypes, and to identify the genes controlling the phenotypes by a map-based cloning strategy. However, it would take a long time and labors to develop NILs because of recurrent backcrossings with marker-assisted selection. In Tomato Genetic Resource Center, University of California, Davis, series of NILs covering the entire genome of LA1777 in the background of *S*. *lycopersicum* E6203 have been registered [59]; however, NILs for chromosome 6 is not available at the time of writing unfortunately. On the other hand, although a group of genetically divergent lines could be useful for the validation, no resistance lines against *P*. *infestans* EG_12 have identified except for *S*. *habrochaites* LA1777 [15]. This meant that this approach might be not suitable for the case of this study.

It should be possible to breed new varieties with high disease resistance by combining the new locus with previously reported genes [19, 20]. Such a ‘gene pyramid’ strategy resulting in durable resistance could contribute to successful management of new populations of *P*. *infestans*, which are resistant not only to well-known R genes, but also to certified fungicides, e.g., metalaxyl [60, 61]. Because we have characterized many *P*. *infestans* isolates [15, 62], as well as tomato wild relatives highly resistant to these isolates [15], further novel resistance loci could be identified from these materials using an approach similar to the one employed in this study.

The genotyping analysis was completed in a short time by taking advantage of two NGS technologies, ddRAD-Seq and WGRS. In the former type of analysis, the number of detectable SNPs depends on genetic diversity (i.e., the so-called genetic distance) of the materials [32, 63, 64]. In this study, because the parental lines were genetically divergent, the number of obtained SNPs was 6,514. This result is consistent with a previous report in which 8,784 SNPs were obtained from an interspecific cross between different species [65]. In intercrossing, or crossing between closely related species, even though the number of SNPs obtained by ddRAD-Seq might be small [66], WGRS has the potential to overcome this issue [43]. Therefore, lab work is no longer a limiting factor in the discovery of new genetic loci.

ddRAD-Seq analysis and WGRS are powerful tools for gene mapping. Previously, it was common to employ SSR and SNP markers for such analysis [28, 29, 67]. However, because these methods are time-consuming and laborious, it used to be difficult to analyze multiple populations at once. Furthermore, even if genetic loci could be narrowed down to small genomic regions, subsequent sequencing of the target regions was necessary for identification of candidate genes of interest. By contrast, ddRAD-Seq analysis can be performed in parallel across multiple mapping populations. In addition, WGRS is the most effective and easiest method for identifying sequence variations in candidate regions. In this study, the alignment rate of the sequence reads to reference sequence was lower in LA1777 than in Castlerock, likely because LA1777 is a wild species belonging to the Eriopersicon subsection, which is distantly associated with cultivated lines such as Castlerock and Heinz 1706 [10].

The distribution patterns of SNPs over the genome was highly biased, with higher density at the distal ends of chromosomes and lower density in pericentromeric regions. This observation was consistent with some previous studies [6, 29, 66] but discordant with another [10]. On the other hand, the density of SNPs identified by the WGRS in this study (512.8 SNPs per 100 kb) was higher than that in a previous study using only cultivated lines (11.9–98.9 SNPs per 100 kb) [33], confirming that wild tomato relatives are genetically distant from cultivated tomato. Thus, it is possible for the WGRS technology to dissect target quantitative traits at nucleotide scale.

Furthermore, WGRS also makes it possible to predict the effects of sequence variations on gene function, which facilitates the identification of candidate genes. In this study, we identified three candidate R genes encoding a nucleotide-binding site leucine-rich repeat (NBS-LRR) protein, a mitogen-activated protein kinase kinase kinase (MAPKKK), and a receptor-like protein kinase (RLK); these gene families are involved in disease resistance and signaling pathways linked to plant innate immunity not only in tomato, but also in other plant species [68–71]. Indeed, outside the candidate region, we identified moderate-impact SNPs in genes encoding serine/threonine-protein kinases. These genes play important roles in disease resistance and biological defense systems, inducing reactive oxygen species (ROS) bursts and stimulating MAP kinases, as demonstrated in *Arabidopsis* [72]. Thus, these genes might confer high disease resistance on LA1777. Furthermore, LA1777 possesses other R genes to many types of biotic stresses [73, 74], because it has not undergone the domestication process, which decreases the level of resistances [75]. The combination of ddRAD-Seq and WGRS could facilitate identification of genes of interest in LA1777. In addition to the genotyping methods, comparative genomics and transcriptomics in tomato and its relatives are useful methods in the post–genome sequencing era [6, 39, 41, 42].

The resolution of genetic mapping depends on the frequency of chromosome recombination in the population, which unfortunately remains uncontrollable. Therefore, even though ddRAD-Seq and WGRS are available, identification of target genes requires fine-mapping. Accordingly, we performed additional DNA marker analysis with SSRs and/or SNPs in the target regions. In the future, due to decreasing sequencing costs for NGS analysis, it will become feasible to perform WGRS across entire mapping populations, not only the parental lines, potentially making fine-mapping with SSR markers and SNPs unnecessary. Disruption of gene functions using genome-editing technologies is also an effective approach for elucidating the functions of genes responsible for target traits.

In conclusion, using the ddRAD-Seq and WGRS NGS technologies, we identified a new resistance locus for late blight disease caused by *P*. *infestans*. DNA markers linked to the locus could be used in MAS in future breeding programs aimed at increasing resistance to this disease. In addition, this approach provides a model for identifying not only additional R genes from tomato relatives and *P*. *infestans* isolates, which our group identified in a previous study [15, 62], but also other genes responsible for desirable agronomical traits. Furthermore, our results confirmed that, as previously reported [15, 58], S. *habrochaites* accession LA1777 represents a useful genetic resource for smart tomato breeding programs, genetics, and genomics studies.

## Supporting information

S1 Fig Physical positions of SNP markers across the tomato chromosomes (Chr00 –Chr12) except ch06 using R package. The SNP markers were generated using the next-generation sequencing technologies and mapped on the reference genome of tomato SL3.0 version, (A) the double-digest restriction site–associated DNA sequencing (ddRAD-Seq), (B) the whole-genome shotgun resequencing (WGRS) approaches.
(PDF)

S1 Table Sequences of oligonucleotides used in ddRAD-Seq. (XLSX)

S2 Table Information of TES and TGS-SSR markers used in the current study. (XLSX)

S3 Table Number of paired-reads and alignment rate of ddRAD-Seq data for F_2_ population mapped onto the tomato reference genome SL3.0. (XLSX)

S4 Table Distribution of the SNP markers on the 12 tomato chromosomes from WGRS and ddRAD-Seq analysis. (XLSX)

S5 Table Number of paired-reads and alignment rate of cv. Castlerock and LA1777 generated from WGRS analysis mapped onto the tomato reference genome SL3.0 version. (XLSX)

S6 Table Putative impact of SNPs on gene functions in the tomato genome of WGRS and candidate regions data. (XLSX)
