# Supplementary material for: Rapid identification of candidate genes for resistance to tomato late blight disease using next-generation sequencing technologies
Source: PLoS One. 2017 Dec 18;12(12):e0189951. doi: 10.1371/journal.pone.0189951 (PMC5734779; doi:10.1371/journal.pone.0189951)
Supplement: S1 Fig — The SNP markers were generated using the next-generation sequencing technologies and mapped on the reference genome of tomato SL3.0 version, (A) the double-digest restriction site–associated DNA sequencing (ddRAD-Seq), (B) the whole-genome shotgun resequencing (WGRS) approaches. (PDF) [file pone.0189951.s001.pdf]

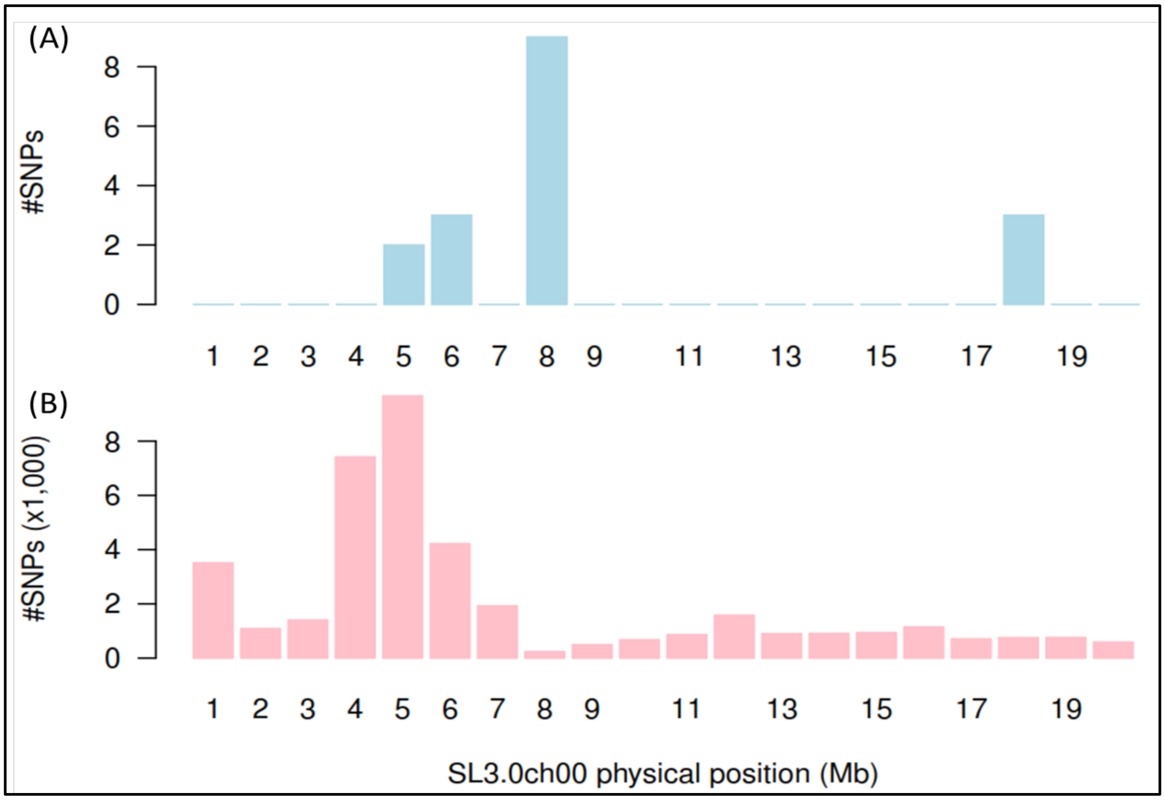
(A)
#SNPs
8
6
4
2
0
1
2
3
4
5
6
7
8
9
11
13
15
17
19
(B)
#SNPs (x1,000)
8
6
4
2
0
1
2
3
4
5
6
7
8
9
11
13
15
17
19
SL3.0ch00 physical position (Mb)

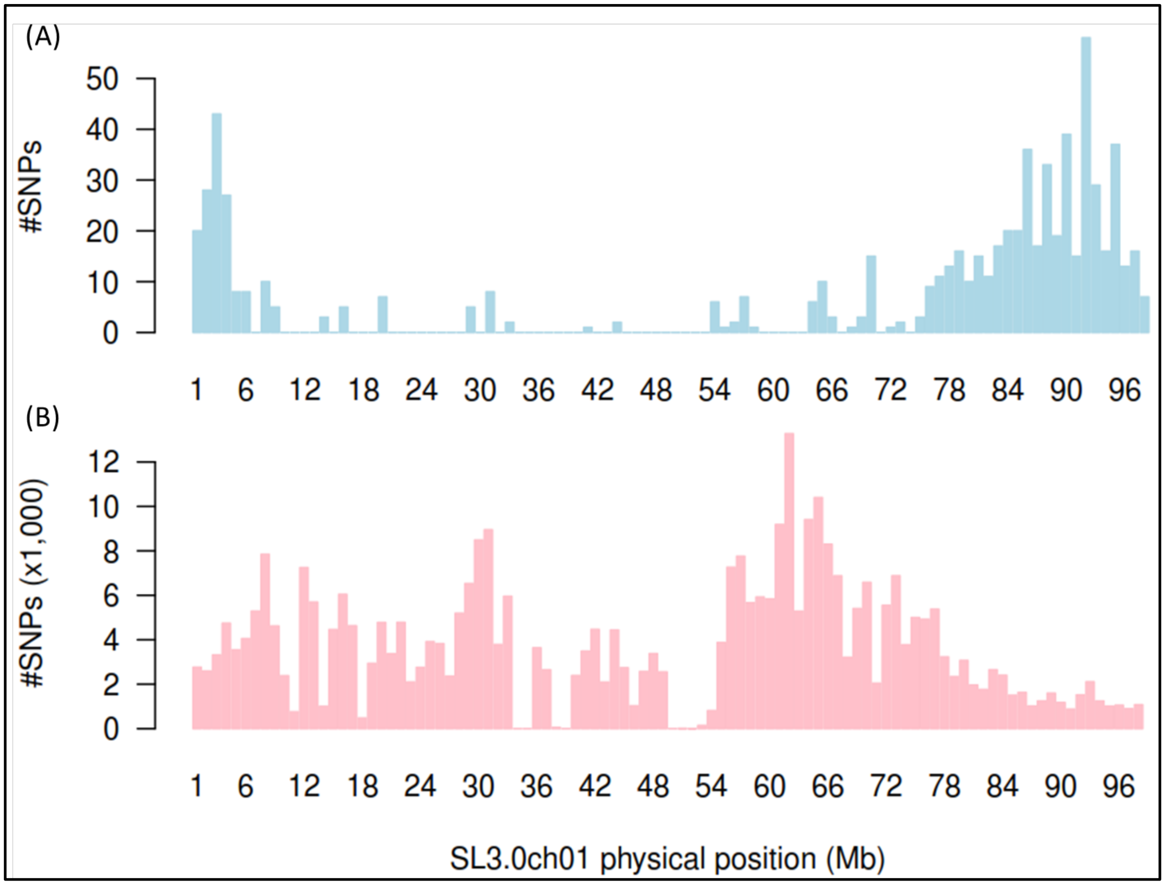
(A)
#SNPs
50
40
30
20
10
0
1
6
12
18
24
30
36
42
48
54
60
66
72
78
84
90
96
(B)
#SNPs (x1,000)
12
10
8
6
4
2
0
1
6
12
18
24
30
36
42
48
54
60
66
72
78
84
90
96
SL3.0ch01 physical position (Mb)

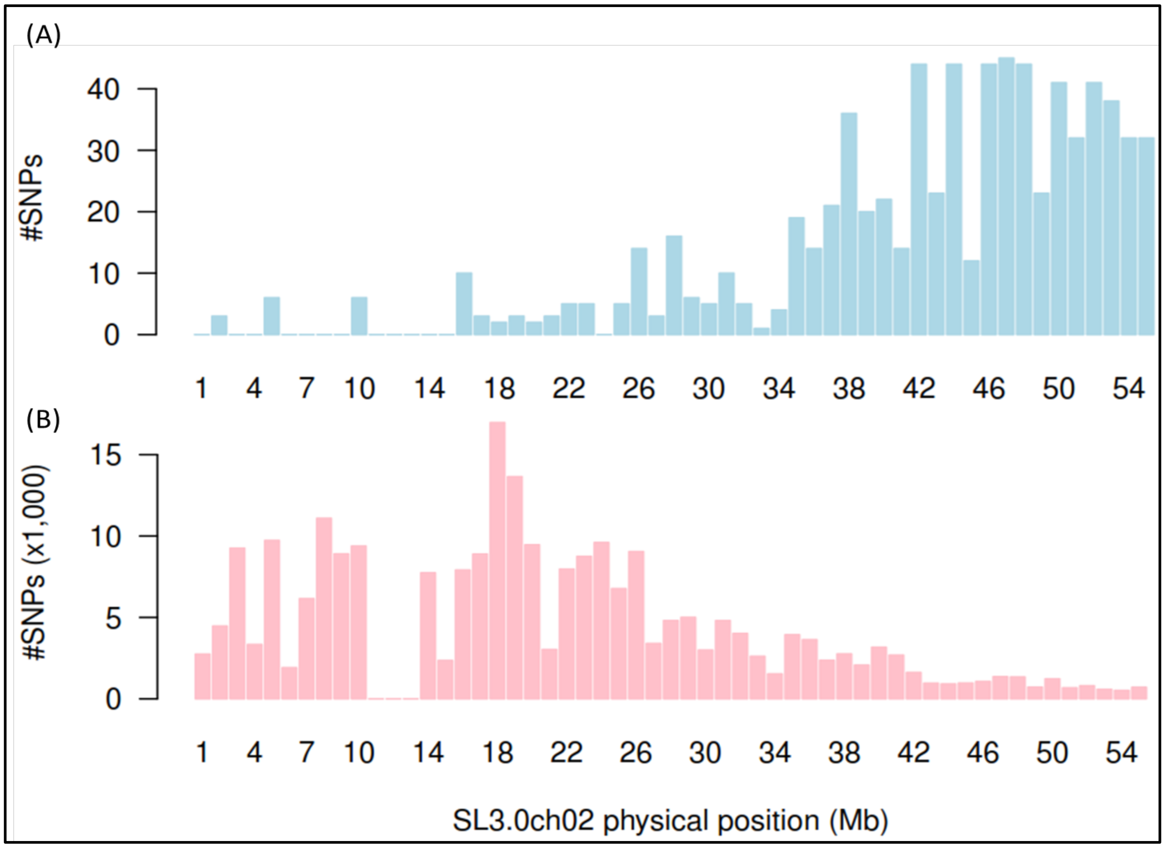
(A)
#SNPs
40
30
20
10
0
1 4 7 10 14 18 22 26 30 34 38 42 46 50 54
(B)
#SNPs (x1,000)
15
10
5
0
1 4 7 10 14 18 22 26 30 34 38 42 46 50 54
SL3.0ch02 physical position (Mb)

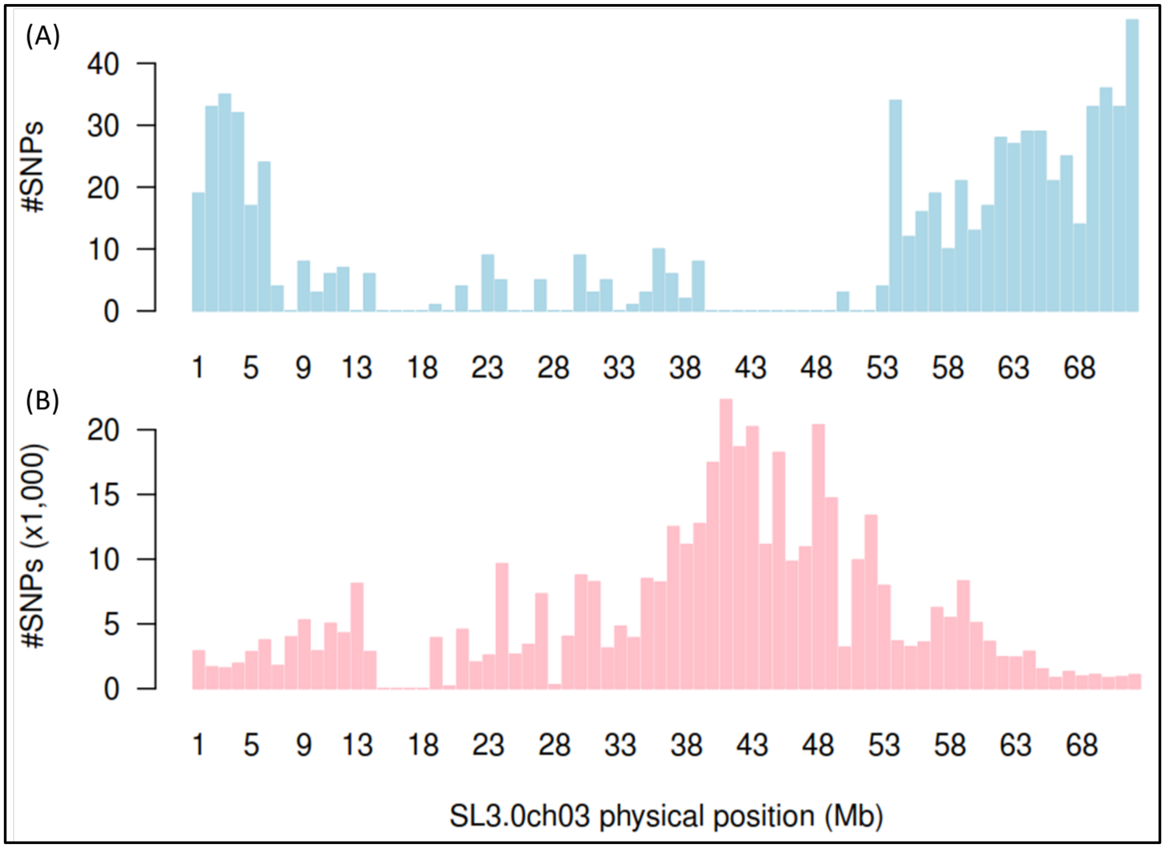
(A)
#SNPs
40
30
20
10
0
1 5 9 13 18 23 28 33 38 43 48 53 58 63 68
(B)
#SNPs (x1,000)
20
15
10
5
0
1 5 9 13 18 23 28 33 38 43 48 53 58 63 68
SL3.0ch03 physical position (Mb)

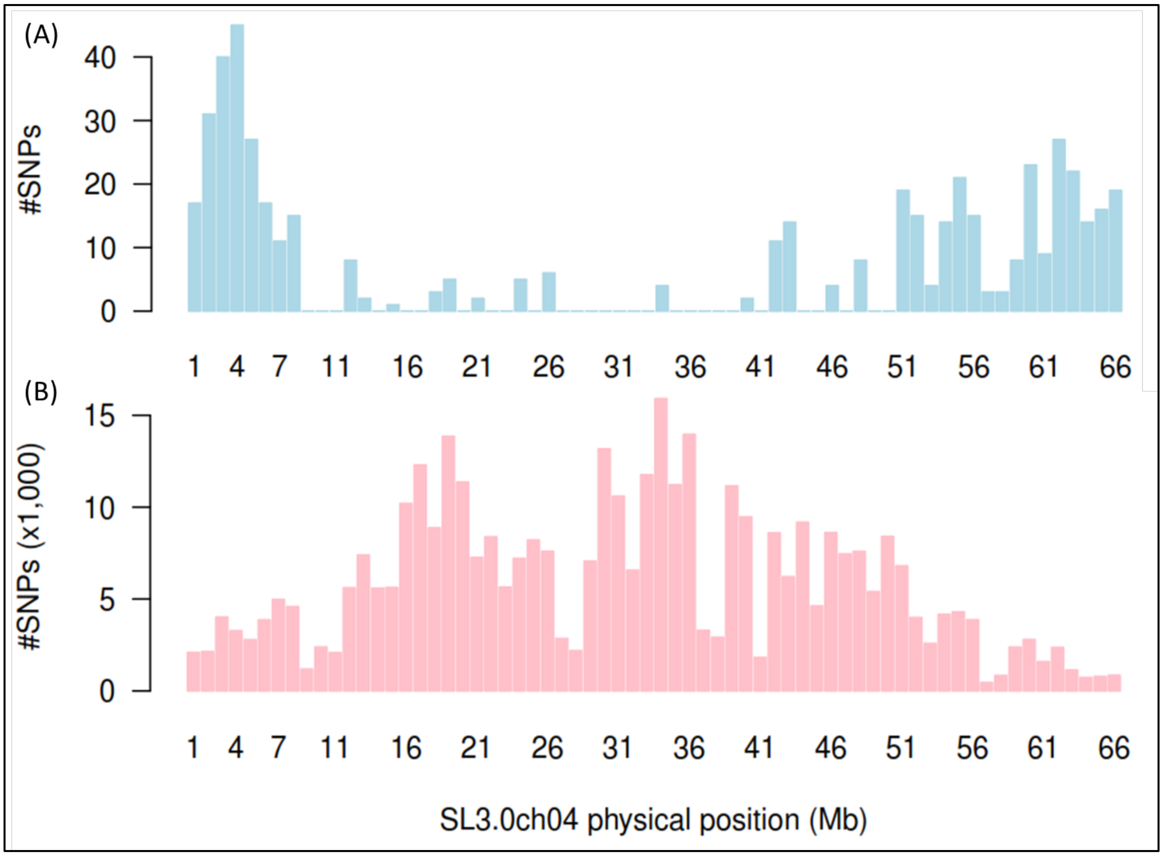
(A)
#SNPs
0
10
20
30
40
1 4 7 11 16 21 26 31 36 41 46 51 56 61 66
(B)
#SNPs (x1,000)
0
5
10
15
1 4 7 11 16 21 26 31 36 41 46 51 56 61 66
SL3.0ch04 physical position (Mb)

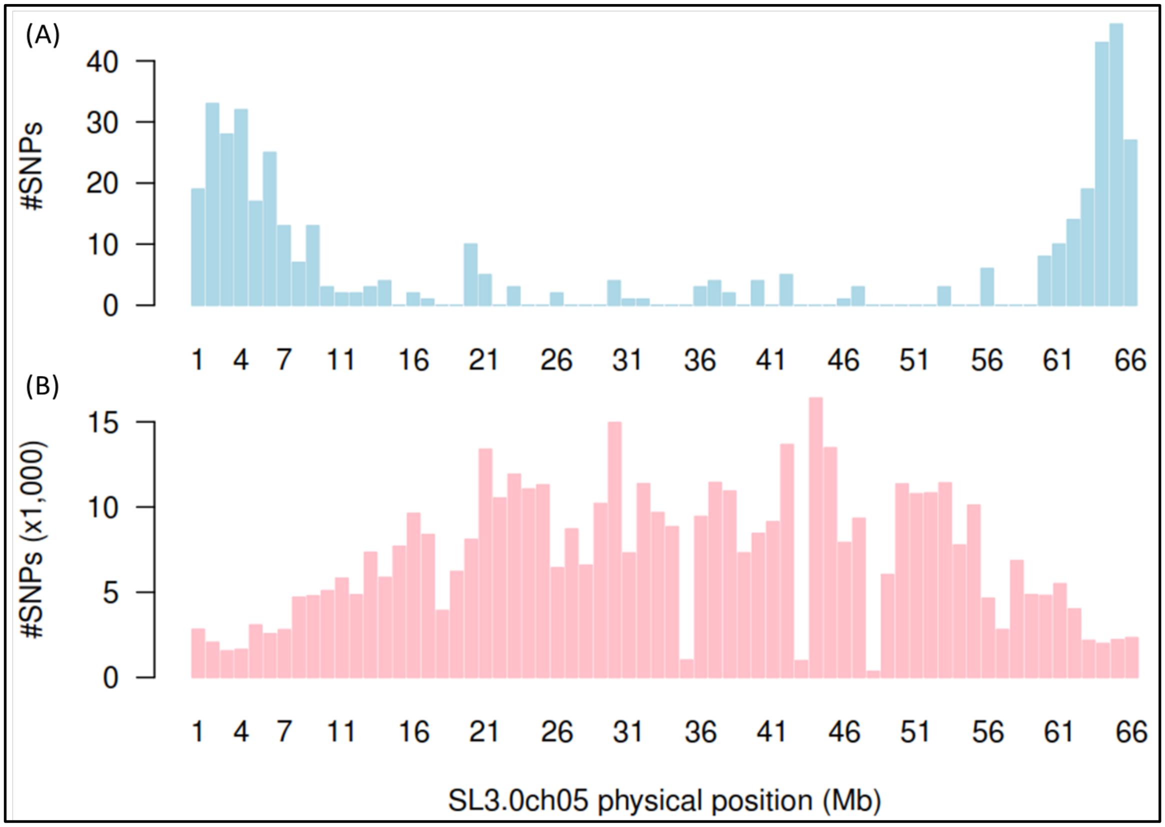
(A)
#SNPs
0
10
20
30
40
1 4 7 11 16 21 26 31 36 41 46 51 56 61 66
(B)
#SNPs (x1,000)
0
5
10
15
1 4 7 11 16 21 26 31 36 41 46 51 56 61 66
SL3.0ch05 physical position (Mb)

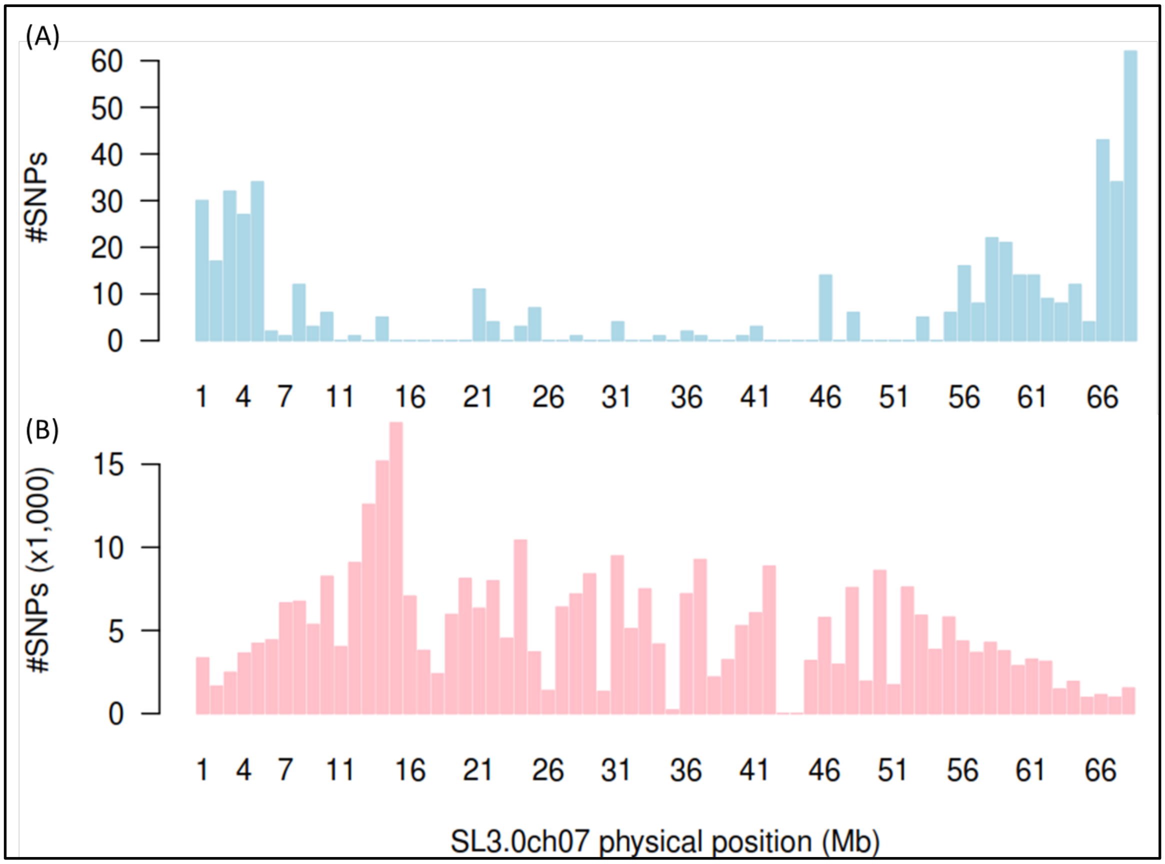
(A)
#SNPs
60
50
40
30
20
10
0
1 4 7 11 16 21 26 31 36 41 46 51 56 61 66
(B)
#SNPs (x1,000)
15
10
5
0
1 4 7 11 16 21 26 31 36 41 46 51 56 61 66
SL3.0ch07 physical position (Mb)

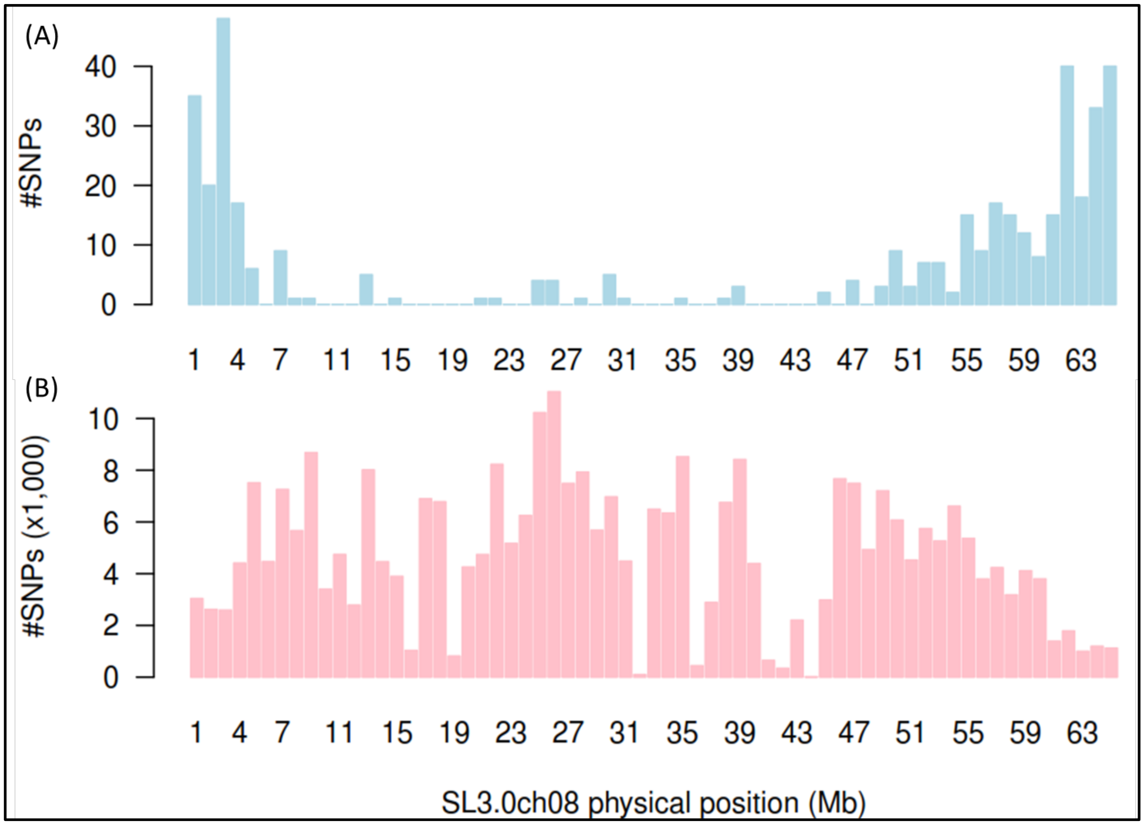
(A)
#SNPs
40
30
20
10
0
1 4 7 11 15 19 23 27 31 35 39 43 47 51 55 59 63
(B)
#SNPs (x1,000)
10
8
6
4
2
0
1 4 7 11 15 19 23 27 31 35 39 43 47 51 55 59 63
SL3.0ch08 physical position (Mb)

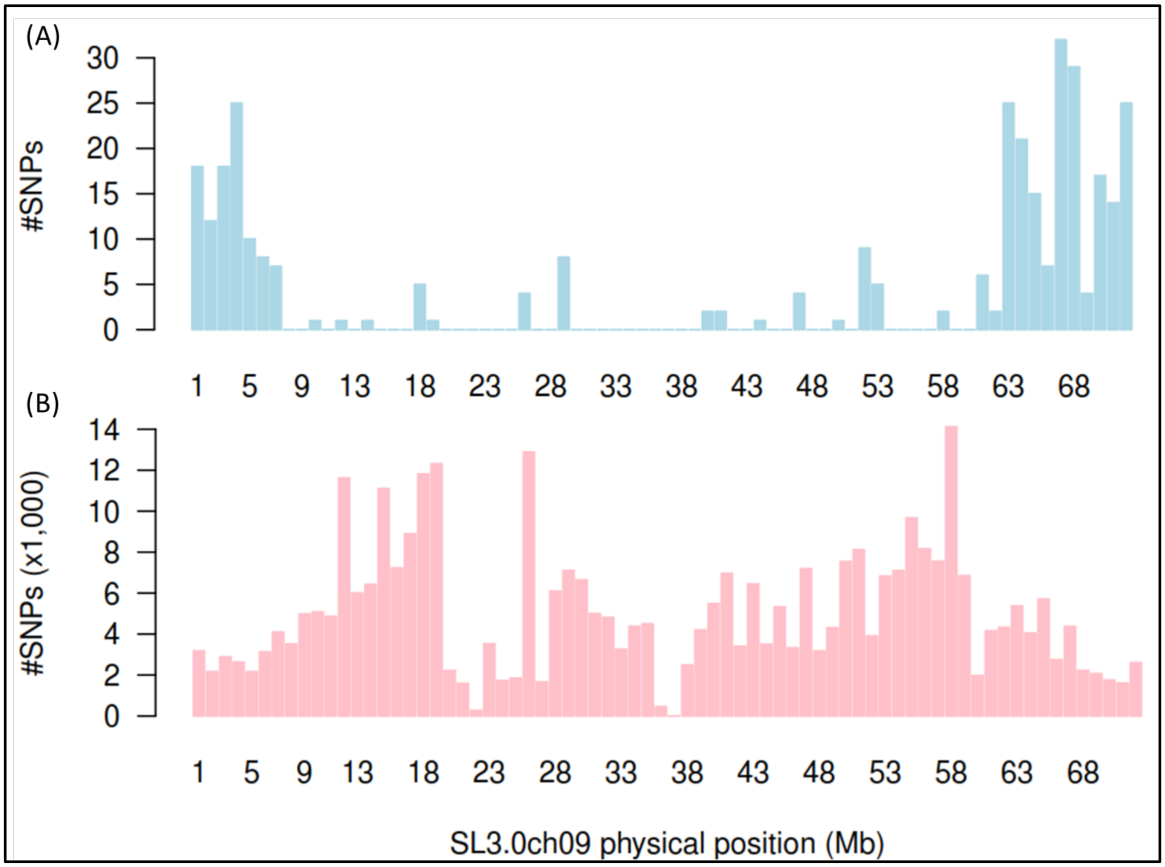

(A)
#SNPs
30
25
20
15
10
5
0
1 5 9 13 18 23 28 33 38 43 48 53 58 63 68
(B)
#SNPs (x1,000)
14
12
10
8
6
4
2
0
1 5 9 13 18 23 28 33 38 43 48 53 58 63 68
SL3.0ch09 physical position (Mb)

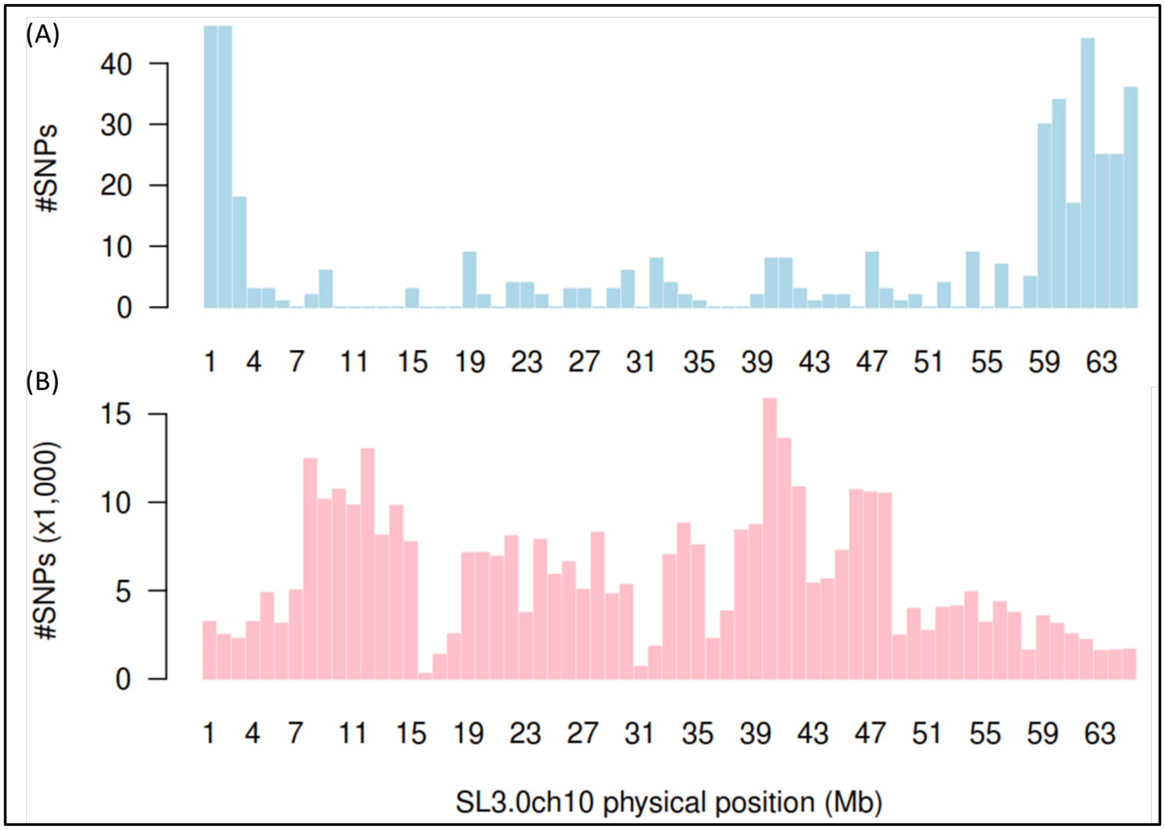

(A)
#SNPs
40
30
20
10
0
1 4 7 11 15 19 23 27 31 35 39 43 47 51 55 59 63
(B)
#SNPs (x1,000)
15
10
5
0
1 4 7 11 15 19 23 27 31 35 39 43 47 51 55 59 63
SL3.0ch10 physical position (Mb)

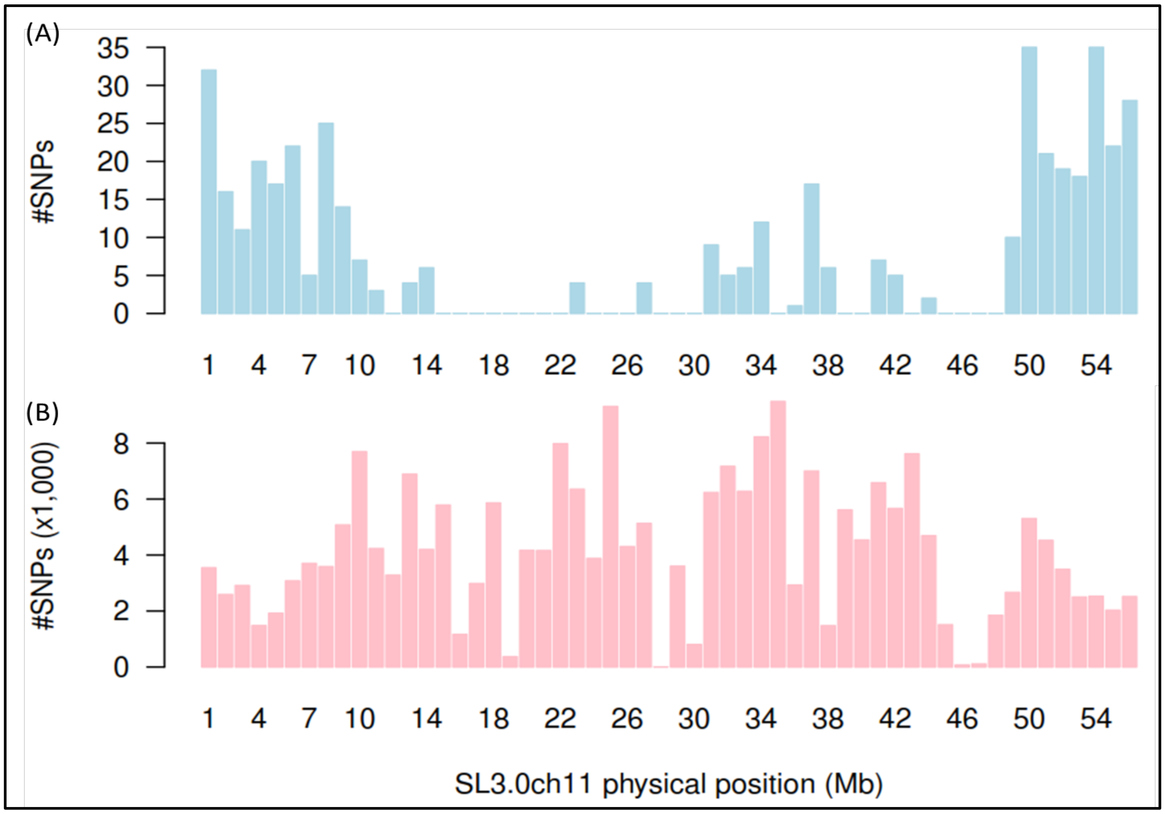

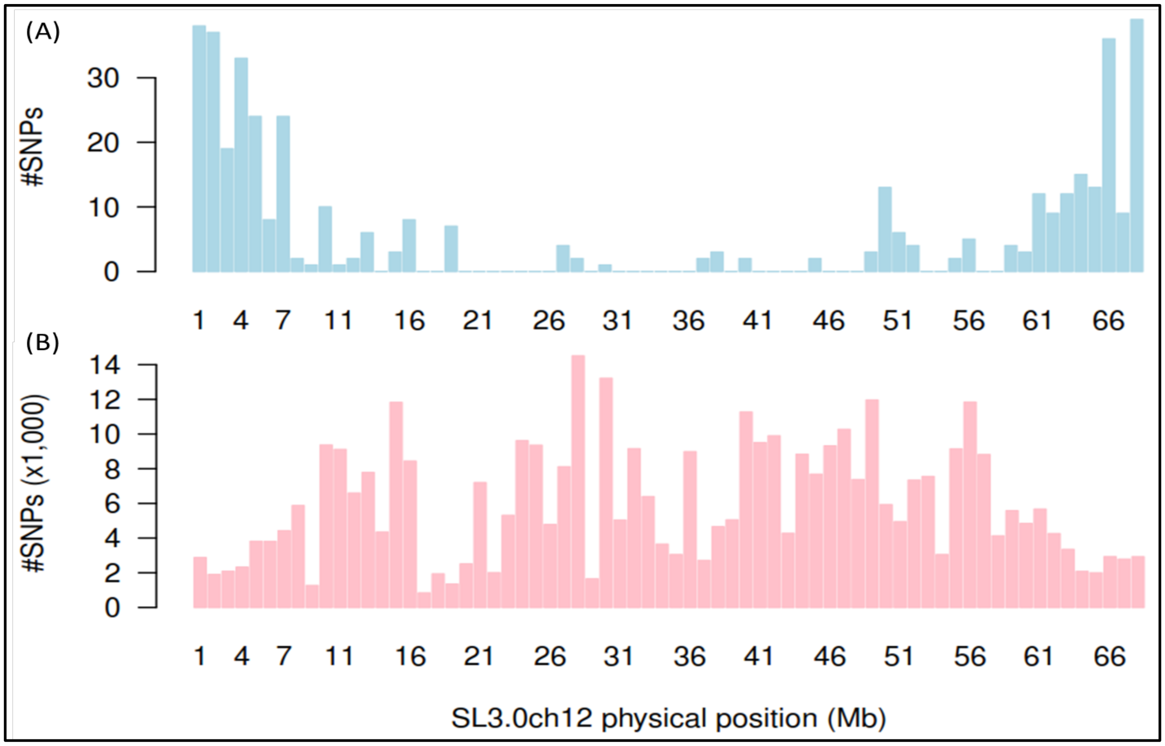

Fig S1. Physical positions of SNP markers across the tomato chromosomes (Chr00 – Chr12) except ch06 using R package. The SNP markers were generated using the next-

generation sequencing technologies and mapped on the reference genome of tomato SL3.0 version, (A) the double-digest restriction site–associated DNA sequencing (ddRAD-Seq), (B) the whole-genome shotgun resequencing (WGRS) approaches.
